# Prevalence, Risk Factors, and Pathophysiology of Dysglycemia among People Living with HIV in Sub-Saharan Africa

**DOI:** 10.1155/2018/6916497

**Published:** 2018-05-23

**Authors:** Benson Njuguna, Jepchirchir Kiplagat, Gerald S. Bloomfield, Sonak D. Pastakia, Rajesh Vedanthan, John R. Koethe

**Affiliations:** ^1^Moi Teaching and Referral Hospital, P.O. Box 4606-30100, Eldoret, Kenya; ^2^Academic Model Providing Access to Healthcare (AMPATH), P.O. Box 4606-30100, Eldoret, Kenya; ^3^Duke Clinical Research Institute, Duke Global Health Institute, Duke University, 2400 Pratt Street, Durham, NC 27710, USA; ^4^Purdue University College of Pharmacy, P.O. Box 5760 Eldoret 30100, Kenya; ^5^Zena and Michael A. Wiener Cardiovascular Institute, Department of Medicine and Department of Population Health Science and Policy, Icahn School of Medicine at Mount Sinai, One Gustave Levy Place, P.O. Box 1030, New York, NY 10029, USA; ^6^Division of Infectious Diseases, Vanderbilt University Medical Center, A2200-MCN 1161 21st Avenue South, Nashville, TN 37232, USA

## Abstract

**Objective:**

To review available literature on the prevalence, risk factors, pathophysiology, and clinical outcomes of dysglycemia among people living with HIV (PLHIV) in sub-Saharan Africa (SSA).

**Methods:**

Database search on PUBMED for eligible studies describing the prevalence, risk factors, pathophysiology, or clinical outcomes of dysglycemia in SSA PLHIV.

**Results:**

Prevalence of diabetes mellitus (DM) and pre-DM among SSA PLHIV ranged from 1% to 26% and 19% to 47%, respectively, in 15 identified studies. Older age and an elevated body mass index (BMI) were common risk factors for dysglycemia. Risk factors potentially more specific to PLHIV in SSA included exposure to older-generation thymidine analogues or protease inhibitors, malnutrition at ART initiation, a failure to gain fat mass on treatment, and elevated serum lipids. There is evidence of higher nephropathy and neuropathy rates among PLHIV in SSA with comorbid DM compared to HIV-negative individuals with DM.

**Conclusion:**

There is a need for longitudinal studies to enhance understanding of the risk factors for dysglycemia among PLHIV in SSA, further research into optimal therapies to reduce pre-DM progression to DM among SSA PLHIV, and studies of the burden and phenotype of diabetic complications and other health outcomes among PLHIV with comorbid DM in SSA.

## 1. Introduction

Sub-Saharan Africa (SSA) accounts for 80% of the global HIV burden and 60% of new HIV infections [1]. Wide-scale adoption of combination antiretroviral therapy (ART) has decreased infection-related mortality among people living with HIV (PLHIV) and increased life expectancy, but this success is tempered by an increasing burden of noncommunicable diseases (NCDs) [2, 3]. Studies of US patient cohorts found PLHIV had as high as a fourfold elevated risk of diabetes mellitus (DM) as compared to HIV-negative persons after adjusting for other risk factors [4, 5]. Current evidence, predominantly from US and European cohorts, indicates this elevated risk of dysglycemia, encompassing both DM and prediabetes (pre-DM; defined as impaired fasting glucose (IFG) or impaired glucose tolerance (IGT)), in PLHIV likely reflects a mix of the effects of HIV per se, chronic inflammation, and some ART agents on glucose metabolism, as well as potentially disproportionate contributions of obesity and older age to DM risk among PLHIV compared to the general population [6, 7].

The majority of studies on dysglycemia in PLHIV come from high-income country settings (HIC), and as a result, the extent to which identified risk factors associated with dysglycemia burden, morbidity, and mortality can be extrapolated to SSA populations is unclear. PLHIV in SSA have several characteristics that may lead to differences in dysglycemia risk compared to PLHIV in HIC. These include (i) higher levels of inflammation biomarkers such as high sensitivity C-reactive protein (hsCRP) and fibrinogen in HIV-negative SSA populations compared to HIC populations potentially reflecting a higher background inflammatory state [8], (ii) ongoing or recent use of older generation ART agents in SSA associated with the development of lipodystrophy and dysglycemia (e.g., thymidine analogues), (iii) limited access to DM screening, prevention, and treatment services in SSA [9], and (iv) a lower prevalence of traditional risk factors for DM such as advanced age, obesity, dyslipidemia, and sedentary lifestyles [10–13].

HIV and dysglycemia are independent risk factors for cardiovascular disease (CVD) and CVD events, such as stroke and myocardial infarction, chronic kidney disease (CKD), neurocognitive decline, and other comorbidities [14–20]. Pre-DM not only predicts future development of DM, with 4–20% of pre-DM progressing to DM annually in the general population if no pharmacological or nonpharmacological interventions are made, but it is also an independent risk factor for CVD [21]. Consequently, knowledge of the risk factors and burden of dysglycemia among SSA PLHIV is crucial in identifying gaps in care and future research priorities. We undertook this review to describe the relatively limited literature on the prevalence, risk factors, pathophysiology, and clinical outcomes of dysglycemia among SSA PLHIV and highlight the research gaps and high-priority areas for future research.

## 2. Methods

We searched PUBMED using the keywords “diabetes,” “insulin resistance,” “glucose intolerance,” “dysglycemia,” “sub-Saharan Africa,” “HIV,” “prevalence,” “pathophysiology,” “risk factors,” “mortality,” “morbidity,” and their related terms. Studies were considered for inclusion if they were original research articles and described any of the following: the prevalence, risk factors, pathophysiology, or clinical outcomes of dysglycemia (CVD-related morbidity and mortality, and microvascular or macrovascular complications) in PLHIV in SSA. Additionally, we screened the reference lists of retrieved articles for other sources. Articles published through August 2017 were considered, with no restriction on the start date. We excluded conference abstracts, narrative or systematic reviews, and articles not in English. Results were summarized descriptively in narrative and tabular form. No additional statistical methods were deployed as we did not pool data.

## 3. Results

### 3.1. Prevalence of Dysglycemia among PLHIV in SSA

**Figure 1:**
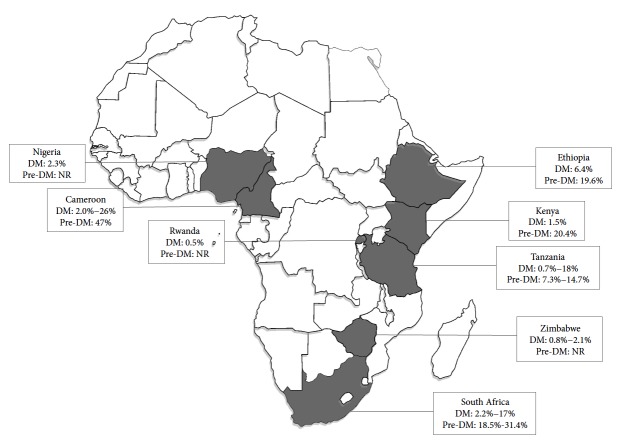
Prevalence of diabetes mellitus (DM) and prediabetes (pre-DM) in HIV-infected patients.

Prevalence data came from 15 studies across 8 countries (Figure 1), highlighting both the dearth of data and variability in the study population. The prevalence of DM among PLHIV ranged from 1% to 26%, while that of pre-DM (IFG or/and IGT) was 19% to 47%, in our reviewed studies [22–36]. There was, however, wide variability in the definition of the population studied, methodology employed, definition of pre-DM, and diagnostic criteria used for DM or pre-DM (Table 1).

Ngatchou et al. reported the highest DM (26%) and pre-DM (47%) prevalence in a cohort of 108 ART-naive PLHIV in Cameroon that was predominantly (74%) female, had a mean age of 39 years, and had a mean BMI of 25.1 kg/m^2^ [34]. Additional characteristics of this population were a mean waist circumference of 81 cm and waist-hip ratio of 0.8, both of which were lower than in HIV-negative controls, who had a much lower prevalence (1%) of DM.

Four studies compared dysglycemia (DM and pre-DM) prevalence between PLHIV on ART versus ART-naive and reported differing results [24, 26–28]. Levitt et al. [27] found progressively higher prevalence of dysglycemia in South African ART-naive PLHIV (22%), PLHIV on 1st line ART (26%), and PLHIV on 2nd line ART (37%). Maganga et al. [28] also reported higher dysglycemia among Tanzanian PLHIV on ART for at least 2 years (33%) compared to ART-naive PLHIV (8%). In contrast, Dave et al. [24] found a nonsignificant difference in dysglycemia prevalence between South African PLHIV on ART for at least 6 months (26%) compared to ART-naive PLHIV (22%) while Kagaruki et al. [26] noted a slightly higher DM prevalence in ART-naive PLHIV (5%) compared to PLHIV on ART (4%). The difference in these findings may partially be explained by the different median durations on ART, for example, 56 versus 16 months in the study by Maganga et al. and Dave et al., respectively, while the duration on ART was not specified in the study by Kagaruki et al.

Five studies compared the prevalence of dysglycemia between PLHIV and HIV-negative controls [22, 28, 32–34]. A trend toward a higher prevalence of dysglycemia among PLHIV was noted compared to HIV-negative individuals although statistically significant differences were found in only two of the studies [28, 34]. A major limitation of the above comparisons was the lack of matching in the control group which led to subsequent between-group differences in potential risk factors for dysglycemia. For instance, in the study by Maganga et al., lower mean age and less central obesity among HIV-negative controls compared to PLHIV at baseline was noted but not adjusted for in the prevalence comparison [28].

### 3.2. Risk Factors for Dysglycemia in PLHIV

Commonly identified risk factors for dysglycemia in our reviewed studies included older age in six studies [23–25, 27, 28, 31] and elevated BMI in three studies [23, 25, 28]. Both age and elevated BMI are considered traditional risk factors for dysglycemia in the general population, and they remain relevant in the PLHIV population [6]. Male gender [23, 24], long-term ART use [28, 31], efavirenz versus nevirapine use [24, 37], and higher CD4 count [24, 28] were identified as risk factors in two studies each. Individual studies found associations between dysglycemia in PLHIV and protease inhibitor use [23], stavudine use [37], zidovudine use [37], and female sex [27]. Of note, we did not find studies in SSA PLHIV that identified an association between inflammation and dysglycemia, yet, from HIC studies, it is evident that markers of inflammation are chronically elevated in both ART-naive and ART-treated PLHIV [38–41] and are potentially related to dysglycemia incidence [42, 43].

### 3.3. Pathophysiology of Dysglycemia in PLHIV in SSA

Obesity prevalence is rising in the general population and among PLHIV in SSA [13, 44, 45], and several studies have demonstrated the steep rise in DM risk accompanying higher BMI values as also reported in US and European cohorts [6, 23, 46–49]. However, there is a clear subset of PLHIV in SSA who develop IFG and DM in the absence of high BMI, though the etiology and the underlying bioenergetics pathway changes of this nonobese DM phenotype are unclear. In South Africa, IFG prevalence was 21% among PLHIV and did not correlate with central obesity [27]. Similarly, BMI did not correlate with insulin sensitivity in a Rwandan PLHIV cohort with a high prevalence of IFG [32]. In a Tanzanian study, the prevalence of DM was over threefold higher in PLHIV compared to HIV-negative individuals, and this difference could not be accounted for by differences in age, gender, BMI, or socioeconomic status [28]. Lastly, in a comparative study in Israel, DM prevalence was higher in Ethiopian immigrant PLHIV (31%) than in native-Israeli PLHIV (4%), with Ethiopians more likely to develop DM at low BMI values [50].

The handful of studies of IFG and DM risk factors among PLHIV in SSA highlights potential pathophysiologic features which may contribute to the development of glucose intolerance in the absence of more widely recognized risk factors, such as obesity or advanced age. Circulating inflammatory cytokine levels are elevated in many PLHIV on ART in SSA, due in part to impaired mucosal defenses, chronic gastrointestinal enteropathy, and opportunistic infections, which may have a role in the development of dysglycemia [51–53]. Prior studies in PLHIV in the US and Europe have linked soluble inflammatory mediators (e.g., C-reactive protein (CRP) and interleukin-6 (IL-6)) to insulin resistance or incident DM [42, 43]. In two large PLHIV cohorts, enrollment CRP and IL-6 levels predicted incident DM several years prior to onset, and each doubling of enrollment IL-6 was associated with an approximately 30% increased risk of developing DM [42]. While prior studies in SSA have found elevated CRP, IL-6, and other markers of systemic inflammation which were associated with increased mortality and cardiovascular disease [54–58], there is a clear need for data on the relationship of inflammation with metabolic comorbidities. Furthermore, caution is warranted in extrapolating findings from US or European cohorts; a recent study of low BMI Zambian and Tanzanian PLHIV found pre-ART and on-ART serum CRP levels did not predict the risk of IFG and DM, though IL-6 and other cytokines were not measured [59].

A study from Ethiopia found elevated low-density lipoprotein (LDL) was independently associated with the development of DM in predominantly non-overweight/obese PLHIV [31]. In contrast, a second study from Ethiopia found total cholesterol (a measurement that incorporates LDL, high-density lipoprotein (HDL), and triglycerides), but not LDL alone, was associated with metabolic syndrome as defined by the National Cholesterol Education Program: Adult Treatment Panel III (ATP) criteria [60]. An association between LDL and ATP-defined metabolic syndrome was also not observed in a large multicenter study of PLHIV at 32 worldwide sites (though no sites in SSA were included) [61]. These conflicting results highlight the need for further investigation of the relationship between hyperlipidemia and glucose tolerance among PLHIV SSA, particularly given the lower BMI often present in this population. Studies early in the HIV epidemic found de novo hepatic lipogenesis was increased over threefold among PLHIV with recent weight loss compared to HIV-negative controls, in addition to accelerated lipolysis and failure to consume plasma free fatty acids [62]. There is evidence that excessive circulating free fatty acids and protein-bound lipids contribute to the development of other metabolic abnormalities. Persistently, high serum lipids are linked to the development of steatohepatitis in PLHIV in SSA and other regions, which can be accompanied by additional ectopic lipid deposits (e.g., intramyocellular or intramyocardial) and glucose intolerance [63–69].

While most patients gain weight after starting ART, particularly those with a lower pretreatment BMI [70, 71], the early recovery of adipose tissue may be unevenly distributed and can evolve into an abnormal repartitioning termed ‘lipodystrophy' [72–79]. Even in the absence of clinically apparent changes, radiographic studies have found a significantly lower percentage of extremity body fat in treated PLHIV compared to healthy controls, suggesting lipodystrophy represents a continuum and most PLHIV remain susceptible to some degree [80]. Several longitudinal studies from SSA demonstrate central fat accumulation and peripheral fat loss among PLHIV on ART [81–83]. The persistence of these changes in body habitus may have been exacerbated by older-generation nucleoside reverse transcriptase inhibitor (NRTI) use in some treatment programs after these agents had been largely replaced in US and European settings. Loss of limb fat, attributed to mitochondrial DNA polymerase *γ* inhibition and impaired respiratory chain efficiency in adipocytes, is more prevalent with older thymidine analogues (e.g., stavudine and zidovudine) compared to newer agents (e.g., lamivudine, abacavir, and tenofovir) [84–86]. Adipose tissue samples from lipoatrophic individuals treated with zidovudine or stavudine demonstrate higher macrophage infiltration and proinflammatory cytokine production; two features thought to contribute to adipocyte insulin resistance and altered lipid handling [87–92].

**Figure 2:**
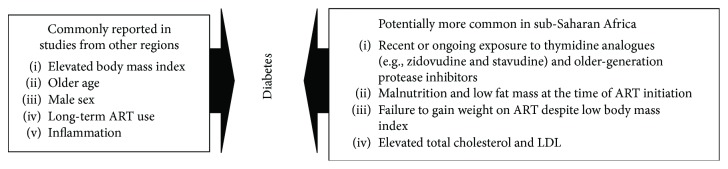
Summary of factors associated with prevalent or incident diabetes in studies of PLHIV from sub-Saharan Africa.

In a recent, large longitudinal study in Zambia and Tanzania of PLHIV who started ART at a low BMI, the risk of developing IFG or DM after treatment initiation was paradoxically *highest* among those with the *lowest* pre-ART hip circumference and body fat mass index [59]. Furthermore, the risk of diabetes was also inversely related to the change in BMI after 2-3 years of ART; patients who started ART with a low BMI and failed to gain weight were at a *higher* risk of dysglycemia after adjusting for multiple other risk factors [59]. These findings suggest that the presence of advanced nutritional wasting at ART start and a lack of nutritional recovery on treatment may predispose to the development of glucose intolerance. Notably, these results are similar to US studies showing lower limb fat in PLHIV is correlated with higher insulin resistance [93, 94]. Further studies are needed to understand whether a combination of poor nutritional status at the time of ART initiation, the demonstrated deleterious effects of some ART agents on adipose tissue function, and potentially other factors could predispose PLHIV in SSA to IFG or DM in the absence of obesity and other common risk factors (Figure 2). The prevalence of IFG and DM is high among PLHIV in SSA, but the marked variability in risk factors observed in prior studies underscores the need to further investigate the range of clinical phenotypes and the accompanying perturbations in bioenergetics pathways in a systematic manner.

### 3.4. Clinical Outcomes among SSA PLHIV with Comorbid Dysglycemia

Data on morbidity and mortality attributable to cardiovascular, microvascular, and macrovascular complications associated with comorbid dysglycemia in SSA PLHIV are scarce. In a study in Malawi of 281 patients with DM, 14% of whom were PLHIV, vision-threatening diabetic retinopathy was not associated with HIV status [95]. In South Africa, Pillay et al. [96] reported significantly higher nephropathy based on proteinuria (26% in PLHIV compared to 16% in HIV-negative) and neuropathy (50% in PLHIV compared to 44% in HIV-negative) in a chort of diabetic persons. This finding may have been related to poorer glucose control among PLHIV on either insulin or oral hypoglycemic agents. In contrast, they found higher prevalence of stage 2 or greater CKD based on KDOQI definitions in the HIV-negative cohort (42%) compared to PLHIV (31%), a finding that the authors partly attributed to the higher age range of the HIV-negative cohort (51–70 years) compared to PLHIV (41–60 years). A major limitation of this study was that HIV status was determined by self-report and was not directly confirmed.

### 3.5. Research Gaps and Priorities

While our review noted numerous gaps in the literature on dysglycemia in SSA PLHIV, we chose to highlight three specific areas that may form research priorities for future investigations. These are (i) the establishment of longitudinal PLHIV cohorts to improve our understanding of the causative associations between various risk factors and dysglycemia incidence, (ii) research into the interruption of progression from pre-DM to DM in SSA PLHIV, and (iii) studies on the clinical outcomes associated with comorbid HIV/DM.

While current studies show overlap between various risk factors that are associated with dysglycemia in SSA PLHIV, such associations remain correlative due to the use of cross-sectional and retrospective study designs in most analyses. An enhanced understanding of the causative risk factors may inform strategies to prevent dysglycemia in PLHIV. There is therefore a need for more longitudinal studies evaluating dysglycemia in SSA PLHIV cohorts. These may take the form of prospective observational studies that begin with a normoglycemic PLHIV cohort and follows them for a long period of time as has been done in some HIC settings [42, 43]. Understudied risk factors such as inflammation need to be evaluated, while the true effect of past and current ART agents, the duration of ART use, and the effect of known traditional risk factors (BMI, anthropometrics, fat distribution and nutrition) requires more investigation in this population.

The range of pre-DM prevalence (19% to 47%) was consistently high across our reviewed studies, representing an opportune area for research into interruption of disease progression in this cohort. Studies from the general population in HIC indicate that rates of pre-DM progression to overt DM may be decreased by 58% through the use of pharmacological interventions and lifestyle modification [97–99]. The extent to which these findings can be extrapolated to PLHIV populations, particularly in SSA, remains unknown. Nevertheless, these studies suggest a potential role for lifestyle interventions, anti-inflammatory therapy, and early use of antidiabetic agents (e.g., metformin) that could be investigated in SSA PLHIV populations.

There is a paucity of data on clinical outcomes among SSA PLHIV with DM, particularly with respect to CVD mortality and morbidity, and microvascular/macrovascular complications of DM and/or HIV. DM and HIV are both CVD risk factors, portending an elevated risk in patients with comorbid HIV/DM. Furthermore, studies suggest that control of DM, and other NCD comorbidities such as dyslipidemia and hypertension, is poorer compared to HIV-negative individuals [96], representing potentially worse outcomes in this cohort.

## 4. Conclusion

The prevalence of DM and pre-DM among PLHIV in SSA ranges from 1% to 26% and 19%–47%, respectively, reflecting an overall high burden of dysglycemia. However, variations in the study population assessed and diagnostic criteria limit firm conclusions. Older age, male gender, and an elevated BMI in the overweight/obese range are commonly associated risk factors for dysglycemia in SSA PLHIV. The interplay between HIV disease, ART, inflammation, and traditional risk factors in the pathophysiology of dysglycemia in SSA PLHIV is yet to be fully understood. There is a need for long-term longitudinal studies to elucidate the role of various risk factors in incident dysglycemia, future research in evaluating interventions to disrupt the progression of pre-DM to overt DM, and clinical outcome studies in comorbid DM/HIV patients in SSA.

## Figures and Tables

**Table 1:** Summary of studies on prevalence and risk factors of diabetes mellitus and prediabetes among PLHIV.

Author(s) and country (reference number)	Study design and population	Dysglycemia definition	Prevalence	Identified independent risk factors^∗^	Comments
Noumegni et al., Cameroon [35]	Cross-sectional: 452 adults age 30–74 years of whom 400 were on ART	DM: FPG ≥7.0 mmol/l on two separate occasions at least 48 hours apart or self-report of taking antidiabetic medicine	DM: 2.0%	BMI ≥ 30 kg/m^2^ associated with insulin resistance: OR 2.28	Patients on ART had significantly higher BMI, waist circumference, waist-hip ratio, obesity, and abdominal obesity compared to those not on ART

Chimbetete et al., Zimbabwe [23]	Retrospective: 4110 PLHIV aged ≥ 16 years starting ART	DM: at baseline, an RBS > 11.0 mmol/l in the presence of DM symptoms or FPG > 7.0 mmol/l or known diagnosis of DM prior to ART initiation	DM: 0.77%	Male gender: aHR 2.31 Age > 40 years: aHR 2.32 BMI > 30 kg/m^2^: aHR 3.1 (all associated with incident dysglycemia)	While this was an incidence study of 4110 PLHIV starting ART, 42 of the 5467 PLHIV in the initial cohort were excluded due to prevalent DM defined as a known diagnosis of DM or DM diagnosed at the baseline visit

Magodoro et al., Zimbabwe [29]	Retrospective: 1033 PLHIV aged ≥ 18 years on ART	Known diagnosis of DM as per patient records	DM: 2.1%	Associations with dysglycemia not reported	Median duration on ART was 5.3 years Case ascertainment was not possible as details on how DM diagnosis had been made was not available

Levitt et al., South Africa [27]	Cross-sectional: PLHIV aged ≥ 18 years in three groups: 393 ART-naive PLHIV, 439 PLHIV on 1st line ART, and 108 PLHIV on 2nd line ART	DM: FPG ≥ 7.0 mmol/l or 2 hr glucose ≥ 11.1 mmol/l IFG: FPG 6.1 mmol/l and <7.0 mmol/l with normal 2 hr glucose IGT: 2 hr glucose ≥ 7.8–11 mmol/l with FBS < 7.0 mmol/l	DM: On 1st line ART: 2.3% On 2nd line ART: 5.6% ART-naive: 3.1% Pre-DM^a^: On 1st line ART: 23.7% On 2nd line ART: 31.4% ART-naive: 18.6%	Age (years): 35–44 (OR 1.82), 45–54 (3.27), and 55–64 (OR 4.75) BMI > 30 kg/m^2^: OR 1.92 Female gender: OR 2.17 1st line ART use: OR 2.47 2nd line ART use: OR 4.1 (all associated with prevalent dysglycemia)	1st line ART regimens comprised dual NRTI plus one NNRTI while 2nd line ART regimens comprised dual NRTI plus a boosted PI A community-based sample group was also included of 880 participants who were not on ART. Dysglycemia prevalence was lower in this group compared to PLHIV groups; however, their HIV status was not known

Isa et al., Nigeria [25]	Retrospective: 2632 ART-naive PLHIV aged ≥ 18 years	DM: RBS ≥ 11.1 mmol/l or FPG ≥ 7.0 mmol/l or self-reported use of antidiabetic drugs	DM: 2.3%	Age > 40 years associated with prevalent dysglycemia: aOR 3.5 BMI ≥ 25 kg/m^2^ associated with incident dysglycemia: aOR 7.5	At one year follow-up after initiating ART, an additional 5.3% of the cohort developed diabetes driving up prevalence to 7.6%

Mohammed et al., Ethiopia [31]	Cross-sectional: 393 PLHIV aged ≥ 21 years of whom 285 were on ART and 109 were ART-naive	DM: FPG ≥ 7.0 mmol/l IFG: FPG ≥ 6.2 mmol/l and <7.0 mmol/l	DM: 6.4% IFG: 19.6%	Age ≥ 40 years: aOR 4.8 ART use ≥ 5 years: aOR 26.93 Hypertension: aOR 4.78 LDL-C ≥ 130 mg/dL: aOR 5.67 (all associated with prevalent dysglycemia)	Lack of OGTT may have underestimated the prevalence of DM and pre-DM

Maganga et al., Tanzania [28]	Cross-sectional: Adults aged > 18 years in three groups: 150 PLHIV on ART for ≥2 years, 151 recently diagnosed ART-naive PLHIV, and 153 HIV-negative	DM: FPG ≥ 7.0 mmol/l or 2 hr glucose ≥ 11.1 mmol/l IFG: FPG 6.1–6.9 mmol/l with normal 2 hr glucose IGT: 2 hr glucose ≥ 7.8–11 mmol/l with FBS < 7.0 mmol/l	DM: On ART: 18% ART-naive: 0.7% HIV (−): 5.2% Pre-DM^a^: On ART: 14.7% ART-naive: 7.3% HIV (−): 2%	ART use ≥ 2 years: aOR 5.72 associated with prevalent dysglycemia	HIV-negative participants were not aged- or sex-matched

Oni et al., South Africa [36]	Retrospective: electronic prescription refill records for 32,474 receiving ≥ 1 prescription for HIV, TB, DM, or/and HTN medications	DM: prescription refill for either metformin, glibenclamide, or insulin	DM: 17%	Associations with dysglycemia not reported	Case ascertainment was not possible as details on how DM diagnosis had been made was not available

Kagaruki et al., Tanzania [26]	Cross-sectional: 671 PLHIV aged ≥ 18 years of whom 354 were on ART and 317 were ART-naive	DM: FPG ≥ 6.1 mmol/l or prior known diagnosis	DM: On ART: 3.7% ART-naive: 4.7%	Associations with dysglycemia not reported	Overall cases of DM were too low to assess between-group difference or associated risk factor relationships Lack of OGTT may have underestimated DM prevalence

Ngatchou et al., Cameroon [34]	Cross-sectional: 108 ART-naive PLHIV and 96 HIV-negative aged-matched controls	IFG: FPG ≥ 5.6–6.9 mmol/lDM: FPG > 6.9 mmol/l	DM: ART-naive: 26% HIV (−): 1% IFG: ART-naive: 47% HIV (−): 27%	Associations with dysglycemia not reported	Dysglycemia prevalence may have been underestimated due to lack of OGTT and exclusion of patients with known, or on treatment for, DM, hypertension or dyslipidemia, cigarette smokers or alcohol users, and patients with a first-degree familial history of DM

Negin et al., South Africa [33]	Survey: 194 PLHIV and 2864 HIV (−) adults aged ≥ 18 years	Self- report of known DM	DM: PLHIV: 4.1% HIV (−): 9.7%	Associations with dysglycemia not reported	Case ascertainment was not possible as DM diagnosis based on self-report Information unavailable for ART use

Dave et al., South Africa [24]	Cross-sectional: 443 PLHIV on ART for ≥6 months and 406 ART-naive PLHIV	DM: FPG ≥ 7.0 mmol/L or 2 hr glucose ≥ 11.0 mmol/l Pre-DM: FPG ≥ 5.6-7.0 mmol/l or 2 hr glucose ≥ 7.8 mmol/l-11.1 mmol/l	DM: On ART: 2.2% ART-naive: 3.4% Pre-DM: On ART: 23.5% ART-naive: 18.5%	Male gender: OR 1.96 Efavirenz use: OR 1.7 All associated with prevalent dysglycemia	Dysglycemia prevalence difference was not statistically significant between on ART and ART-naive group and may be underestimated by the exclusion of known history of DM or IGT ART regimen in use was stavudine or zidovudine with lamivudine and nevirapine or efavirenz Older age (OR 1.04) and CD4 count (OR 1.001) also associated with prevalent dysglycemia but cutoffs not specified

Anastos et al., Rwanda [22]	Cross-sectional: women aged ≥ 25 years divided into two groups: 606 ART-naive PLHIV and 218 HIV-negative	DM: FPG > 6.9 mmol/l or self-reported history of DM	DM: ART-naive PLHIV: 0.5% HIV (−): 0.5%	Associations with dysglycemia not reported	This analysis was based on the Rwanda Women's Inter-association Study and Assessment and inclusion was based on the availability of fasting lipoprotein levels and not glucose levels

Manuthu et al., Kenya [30]	Cross-sectional: 134 PLHIV on ART for ≥4 weeks and 161 ART-naive PLHIV	DM: FPG ≥ 7.0 mmol/l or 2 hr glucose ≥ 11.0 mmol/l IFG: FPG ≥ 6.1 to 6.9 mmol/l IGT: 2 hr glucose ≥ 7.8 mmol/l–11.1 mmol/l	DM: 1.5% Pre-DM^a^: 20.4%	No significant associations with dysglycemia reported	Excluded patients with known DM status thus may underestimate prevalence

Mutimura et al., Rwanda [32]	Cross-sectional: 150 PLHIV on ART for ≥6 months and 50 HIV (−) controls	Dysglycemia: IFG > 5.6 mmol/l	PLHIV: With LDS: 18% Without LDS: 16% HIV (−): 2%	Associations with dysglycemia not reported	Distinction was not made between DM and prediabetes

ADA: American Diabetes Association; aHR: adjusted hazard ratio; aOR: adjusted odds ratio; ART: antiretroviral therapy; FPG: fasting blood glucose; HIV: human immunodeficiency virus; HTN: hypertension; IGT: impaired glucose tolerance; LDL: low-density lipoprotein; LDS: lipodystrophy; NNRTI: nonnucleoside reverse transcriptase inhibitor; NRTI: nucleoside reverse transcriptase inhibitor; OGTT: oral glucose tolerance test; OR: odds ratio; PI: protease inhibitor; PLHIV: people living with HIV; PY: person-years; DM: diabetes mellitus; TB: tuberculosis. ^∗^Only statistically significant risk factors are reported. ^a^Prediabetes definition: impaired fasting glucose or impaired glucose tolerance.
